# Cefditoren: Comparative efficacy with other antimicrobials and risk factors for resistance in clinical isolates causing UTIs in outpatients

**DOI:** 10.1186/1471-2334-12-228

**Published:** 2012-09-25

**Authors:** Despina Hatzaki, Garyphallia Poulakou, Ioannis Katsarolis, Niki Lambri, Maria Souli, Ioannis Deliolanis, Georgios K Nikolopoulos, Evangelia Lebessi, Helen Giamarellou

**Affiliations:** 1 Department of Microbiology, “P. and A. Kyriakou” Children’s Hospital, Thibon and Levadeias, 11527, Athens, Greece; 2 4th Department of Internal Medicine and Infectious Diseases Research Laboratory, Athens University School of Medicine, 1, Rimini St, 12462, Haidari, Athens, Greece; 3 Department of Microbiology, “Laikon” General Hospital of Athens, 17 Agiou Thoma St, 11527, Athens, Greece; 4 Hellenic Centre for Disease Control and Prevention, 3-5 Agrafon St, 15123, Maroussi, Athens, Greece; 5 6th Department of Internal Medicine, Hygeia Hospital, 4 Erythrou Stavrou st and Kifissias Ave, 15123, Maroussi, Athens, Greece

**Keywords:** Urinary tract infections, Acute uncomplicated cystitis, Cephalosporins, Antimicrobial resistance, Empirical treatment

## Abstract

**Background:**

To investigate a possible role of Cefditoren, a recently marketed in Greece third-generation oral cephalosporin in urinary infections of outpatients.

**Methods:**

During a multicenter survey of Enterobacteriaceae causing UTIs in outpatients during 2005–2007, Cefditoren MICs were determined by agar dilution method in a randomly selected sample of uropathogens. Susceptibility against 18 other oral/parenteral antimicrobials was determined according to Clinical and Laboratory Standards Institute methodology.

**Results:**

A total of 563 isolates (330 *Escherichia coli*, 142 *Proteus mirabilis* and 91 *Klebsiella* spp) was studied; MIC50/MIC90 of Cefditoren was 0.25/0.5 mg/L respectively, with 97.1% of the isolates being inhibited at 1 mg/L. All 12 strains producing ESBLs or AmpC enzymes were resistant to cefditoren. Susceptibility rates (%) for amoxicillin/clavulanic acid, cefuroxime axetil, cefotaxime, ciprofloxacin, trimethoprim/sulfamethoxazole and fosfomycin were 93.1- 94.1- 96.8-93.1-71.9 and 92.8% respectively. Cefditoren MIC was significantly higher in nalidixic/ciprofloxacin non-susceptible strains; resistance to cefditoren was not associated with resistance to mecillinam, fosfomycin nitrofurantoin and aminoglycosides. Multivariate analysis demonstrated history of urinary infection in the last two weeks or three months as risk factors for cefditoren resistance.

**Conclusions:**

Cefditoren exhibited enhanced in vitro activity against the most common uropathogens in the outpatient setting, representing an alternative oral treatment option in patients with risk factors for resistance to first-line antibiotics.

## Background

Urinary tract infections (UTIs) represent the most frequent bacterial infection encountered in the community setting being caused in their vast majority by members of the family of Enterobacteriaceae [1,2]. Recently, antimicrobial resistance among uropathogens causing uncomplicated cystitis has increased, as well as the recognition of the importance of the ecological adverse effects of antimicrobial therapy (collateral damage) [2-6]. Effective empirical therapy must be based on susceptibility profiles of the uropathogens, therefore surveillance studies are important tools to guide antibiotic selection [3]. However, in a “real-life” scenario, empirical therapy is most likely to be prescribed either without a urine culture or before the results become available. A recognized drawback of many published studies is the inclusion of strains collected from hospitals, thus limiting surveillance in populations with easy access to tertiary centers even as outpatients. Additionally, in several laboratory-based studies no distinction could be safely made between complicated and uncomplicated UTIs; that same is true for purely “community” infections and nosocomial or healthcare-associated infections that are being treated in the community [3,6].

Cefditoren is a third generation oral cephalosporin with a broad spectrum of activity comprising Gram-positive and Gram-negative bacterial species [7,8]. After an oral 400-mg single dose, the mean concentrations in urine are 186.5 mg/L at 2 to 4 h and 12.7 mg/L at 8 to 12 h; available data have shown its potential to be used in the treatment of UTIs [9]. Cefditoren retained activity against some clinically significant pathogens harboring β-lactamase enzymes. In a study by Sevillano et al., cefditoren exhibited bactericidal activity (>4 log10 reduction) against TEM-1 (penicillinase production or hyperproduction) and TEM-34 derivative (IRT-6) isolates from 4 to 24 h. However, against ESBL-producing strains, no sustained bactericidal activity was demonstrated: against strains harboring the SHV determinant bactericidal activity was achieved only in the 6- to 8-h whereas against the TEM-116 strain a 2-log10 regrowth occurred from 12 to 24 h [9].

The present study was conducted in order to investigate a possible role of cefditoren in the treatment of UTIs treated in the outpatient setting. Given the expanded spectrum of cefditoren, strains of uncomplicated as well as complicated UTIs were included.

## Methods

### Study period and participating institutions

From January 2005 to March 2007 a Greek multicentre surveillance network was formed by private or public microbiology laboratories, representative of the whole country, in order to obtain Enterobacteriaceae isolates from outpatients with UTI. Personal data were collected anonymously. The research protocol was approved by the Ethics Committees of the cooperating hospitals, namely “Attikon” University General Hospital of Athens, “Sismanoglion” General Hospital of Athens and “G. Gennimatas” General Hospital of Athens. Primary care centers and Private Institutions participating in this study do not have Ethics Committees. Isolated strains were shipped to the central laboratory (Laboratory for Infectious Diseases and Antimicrobial Chemotherapy, 4th Dept of Internal Medicine, Athens University School of Medicine, University General Hospital ATTIKON). The network’s structure and methodology have been reported previously [10].

### Bacterial isolates

A single urine culture per outpatient referred for a urine sample culture to each collaborating centre was collected along with a detailed questionnaire tracking demographic and clinical information. Clinical data included: reason for giving a urine sample, symptoms, history of UTI (in the last 2 weeks, 3 months or in the previous year prior to sampling), recent use of antibiotics (in the previous 2 weeks and 3 months), history of admission to the hospital and/or insertion of a urinary catheter during the previous year, presence of diabetes mellitus, nephrolithiasis, presence of urinary catheter and pregnancy on sampling [10]. A culture was considered positive with a growth of a single microorganism >10^4^ CFU/ml. Bacterial isolates were identified by biochemical profiling using API systems (BioMerieux, Basingstoke, UK).

Isolates included in the current study were randomly selected from the total collection, which included 2446 microorganisms, 2280 of them being either *E. coli*, or *Proteus mirabilis* or *Klebsiella* spp. As defined by the study protocol every fourth isolate per center was selected for the cefditoren study; 7 isolates were dropped out due to contamination.

### Antimicrobial susceptibility testing

Antimicrobial susceptibility testing to all antibiotics except Cefditoren was performed using the disk diffusion method, according to the Clinical Laboratory Standard Institute recommendations, CLSI 2011 [11]. Antimicrobials tested (disks) were: Ampicillin (10 μg), Cephalothin (30 μg), Cefuroxime (30 μg), Cefotaxime (30 μg), Ceftazidime (30 μg), Amoxicillin/Clavulanic acid (20/10 μg), Piperacillin/tazobactam (100/10 μg), Ticarcillin/clavulanate (75/10 μg), Imipenem (10 μg), Ciprofloxacin (5 μg), Trimethoprim/Sulfamethoxazole (1.25/23.75 μg), Nalidixic acid (30 μg), Nitrofurantoin (300 μg), Fosfomycin (200 μg), Mecillinam (10 μg), Gentamicin (10 μg), Amikacin (30 μg), Netilmicin (30 μg). Additionally, in view of the widespread use of quinolones in our community, MICs for Ciprofloxacin were determined by E-test method (AB-Biodisk, Solna, Sweden), in order to obtain a depiction of resistance trends within the susceptibility range in our population.

Susceptibility to Cefditoren was tested by agar dilution method, according to CLSI 2011 methodology. *Escherichia coli* ATCC 25922 was used as a quality control microorganism and was included in each run. Cefditoren standard powder was supplied by GlaxoSmithKline (Hellas). In lack of CLSI or EUCAST established breakpoints for Cefditoren, isolates with MIC ≤ 1 mg/L were considered as sensitive according to the recent literature [12-14]. This choice was further supported by the recent EUCAST clinical breakpoints for third generation cephalosporins against Enterobacteriaceae [15].

Phenotypic identification of extended-spectrum β-lactamases (ESBL) production was confirmed following the CLSI (2011) guidelines [11]. Phenotypic identification of plasmidic AmpC β-lactamases was additionally tested using the phenyl boronic acid inhibition method, as described previously [16]. Other mechanisms of resistance (production of inhibitor-resistant TEM [IRT] β-lactamases and penicillinase production or hyperproduction) were detected by phenotypic analysis and interpretation following the EUCAST guidelines [15].

### Definitions

Male gender, pregnancy, history of urinary tract infection in the last two weeks, history of admission to the hospital in the last 30 days, the presence of diabetes mellitus or nephrolithiasis and the presence of a urinary catheter on sampling were considered as complicating factors [10].

Female non pregnant patients, without complicating factors, presenting with at least one urinary symptom (i.e. frequency, dysuria, hematuria, suprapubic pain, excluding fever and vaginal symptoms), and a positive urine culture were assigned to the group “Acute Uncomplicated Cystitis” (AUC) [3].

### Statistical analysis

In the univariate analysis proportions of NS (non susceptible) or R (resistant) strains between categorical variables were compared using the Pearson’s chi-square test or the Fischer’s exact test where appropriate. A p-value <0.05 was considered as statistically significant. Univariate predictors with p < 0.1 were tested for inclusion in a step-wise multivariate model. Nonsignificant variables were removed sequentially until only those significant at p < 0.1 remained. The analysis was carried out using Stata 10.0 (Stata Corp, Texas, USA).

## Results

### Demographic analysis

Cefditoren was studied against a total of 563 isolates (330 *E. coli*, 142 *Proteus mirabilis* and 91 *Klebsiella* spp-three *K. oxytoca* and 88 *K. pneumoniae*). Fully evaluable clinical information was available for 334/563 isolates (59.3%), i.e. 214/330 *E. coli* (64.8%), 75/142 *P. mirabilis* (52.8%) and 45/91 *Klebsiella* spp (49.5%). Women accounted for 88.3% of the patients with a mean age of 45.8 years (SD ± 18.5ys). A total of 318 cases of acute uncomplicated cystitis were available for evaluation. Demographic and clinical data are listed in detail in Table 1.

**Table 1:** Demographic and clinical data per type of infection (for 334 cases out of the total 563 studied)

	**Pts with AUC (%)**	**Total patients sample with available clinical information**
No of patients (% of total)	172 (51.5)	334 (100)
Gender (% female)	100	88.3
Age (mean ± S.D.) (range) (years)	42.6 ± 17.0 (16–93)	46.9 ± 18.6 (16–93)
15–65	141 (82.0)	248 (74.3)
>65	25 (14.0) *	70 (20.9)
Missing	7 (4.0)	16 (4.8)
History of previous UTI in the last 2 weeks	0^a^	13 (3.9)
History of admission in the last year	11 (6.4) *	39 (11.7)
History of urinary catheter placement during admission	7 (4.1)	18 (5.4)
Diabetes mellitus	0^a^	30 (9.0)
Nephrolithiasis	0^a^	27 (8.1)
Actively having a urinary catheter	0 ^a^	15 (4.5)
Pregnancy	0^a^	6 (1.8)
History of UTI in the last 3 months	16 (9.3) *	51 (15.3)
History of UTI in the past	64 (37.2)	122 (36.5)
History of receiving an antibiotic in the last 3 months for reason other than UTI	33 (19.2)	62 (18.6)

*AUC* acute uncomplicated cystitis, *S.D.* standard deviation, *UTI* urinary tract infection.

^a^ Not applicable by protocol definition. *P ≤ 0.05.

### Antimicrobial susceptibility testing for the total sample population

**Figure 1:**
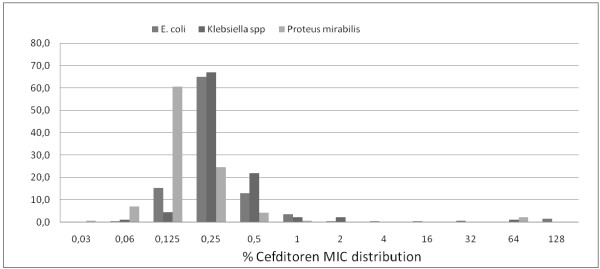
Cefditoren (%) MIC distribution per species.

The in vitro activity of the antimicrobial agents tested against all the isolates in the study are listed in detail in Table 2, in terms of non-susceptibility rates (intermediate resistance and resistance rates). Cefditoren non-susceptibility rates for *E. coli*, *P. mirabilis* and *Klebsiella* spp were 10/330 (3%), 3/142 (2.1%), and 3/91 (3.3%) respectively, while respective values for MIC50/MIC90 were 0.25/0.50, 0.125/0.25, and 0.25/0.5 mg/L (in total 0.25/0.5 mg/L, range 0.03-128 mg/L). In Figure 1, MIC distribution for cefditoren against all isolates of *E. coli, P. mirabilis* and *Klebsiella* spp is displayed.

**Table 2:** **Non-susceptibility **^**a**^**rates (%) for the total isolate yield of the study (n = 563)**

**Antimicrobial agent**	**Escherichia coli (n = 330)**	**Klebsiella spp (n = 91)**	**Proteus mirabilis (n = 142)**
Amoxicillin	31.5	N/A ^b^	33.1
Amoxicillin/clavulanic acid	6.4	1.1	4.2
Cefalothin	9.1	3.3	13.4
Cefuroxime sodium	2.7	1.1	1.4
Cefuroxime axetil	3.9	3.3	2.8
Co-trimoxazole	23.6	11.0	13.4
Nalidixic acid	8.2	9.9	5.6
Ciprofloxacin	4.8	5.5	1.4
Mecillinam	3.6	1.1	22.5
Nitrofurantoin	6.4	30.8	N/A ^b^
Fosfomycin ^c^	1.2	1.1	9.9
Cefotaxime	3.0	1.1	1.4
Ceftazidime	0.6	1.1	3.5
Gentamicin	2.1	1.1	4.2
Netilimicin	0.6	1.1	0.7
Amikacin	0.9	1.1	0
Piperacillin-tazobactam	0.6	1.1	0
Ticarcillin-clavulanate	0.9	1.1	0.7
Imipenem	0.6	0	0.7
Cefditoren	3.0	3.3	2.1

^a^ CLSI 2011 breakpoints of susceptibility were applied.

^b^ N/A: not applicable (species inherently resistant).

^c^ Fosfomycin trometamol is no long marketed in Greece.

Phenotypic identification of ESBL production was evident in 9 strains (8 *E coli*, *1 Klebsiella* spp), while plasmid-mediated Amp-C in 3 strains (3 *P. mirabilis*). Production of inhibitor-resistant TEM β-lactamases and penicillinase production/hyperproduction was identified in 11 strains (9 *E coli*, 2 *Klebsiella* spp). All ESBL-producer strains were resistant to cefditoren (MIC range 16–128 mg/L); the same applied for all three plasmidic AmpC producers possessing MIC of cefditoren at 64 mg/L. TEM/hyperproducers displayed more variable MICs against cefditoren; in 7/11 the MIC was equal to 1 mg/L, whereas it ranged between 2–4 mg/L for the 4 remaining strains of this kind.

Cefditoren displayed good in vitro activity against *E. coli* isolates with different mechanisms of resistance. For ampicillin non-susceptible strains (104 strains) cefditoren MIC50/MIC90 values were 0.25/1 mg/L, whereas for amoxicillin-clavulanate and ciprofloxacin non-susceptible strains (21 and 16 strains respectively) the respective values were 0.5/128 mg/L. Finally, cefditoren MIC50/MIC90 values of cotrimoxazole non-susceptible strains (n = 78) were 0.25/4 mg/L.

### Antimicrobial susceptibility testing for the study population with available clinical data

Non-susceptibility rates for *E. coli* isolates from AUC cases are listed in Table 3. MIC50/90 for cefditoren were 0.25/0.50 mg/L for AUC *E coli* isolates. Non-susceptibility rates for *Proteus mirabilis* and *Klebsiella* spp. isolates from AUC cases are listed in Table 4; MIC50/90 values for cefditoren were 0.125/0.25 and 0.125/0.25 mg/L respectively.

**Table 3:** ***Escherichia coli *non-susceptibility rates **^**a**^**for cases with available clinical data**

**Antimicrobial agent**	**AUC (n = 119)**	**Total*E. coli*cases with available clinical info (n = 214)**
Amoxicillin	26.1	27.6
Amoxicillin/clavulanic acid	5.0	4.7
Cefalothin	6.7	7.9
Cefuroxime sodium^b^	0.8	1.4
Cefuroxime axetil^c^	2.5	3.3
Co-trimoxazole	18.5	18.7
Nalidixic acid	5.0	7.9
Ciprofloxacin	1.7	4.2
Mecillinam	2.5	2.8
Nitrofurantoin	7.6	7.0
Fosfomycin ^d^	0	0.5
Cefotaxime	0.8	2.3
Ceftazidime	0	0
Gentamicin	0	1.4
Netilimicin	0	0.5
Amikacin	0	0
Piperacillin-tazobactam	0	0
Ticarcillin-clavulanate	0.8	0.9
Imipenem	0	0
Cefditoren	1.7	1.9

AUC. acute uncomplicated cystitis; ^a^ CLSI 2011 breakpoints of susceptibility were applied; ^b^Results reported according to the breakpoint of parenteral cefuroxime; ^c^ Results reported according to the breakpoint of oral cefuroxime; ^d^ Fosfomycin trometamol is no long marketed in Greece.

**Table 4:** ***Proteus mirabilis *. and *Klebsiella *spp non-susceptibility rates **^**a**^**for cases with available clinical data**

**Antimicrobial agent**	***Proteus mirabilis***		***Klebsiella *spp.**	
	**AUC**^**b**^**(n = 31)**	**Total cases with available clinical information (n = 75)**	**AUC**^**b**^**(n = 22)**	**Total cases with available clinical information (n = 45)**
Amoxicillin	35.5	33.3	N/A ^c^	N/A ^c^
Amoxicillin/clavulanic acid	6.5	5.3	0	2.2
Cefalothin	12.9	12.0	4.5	4.4
Cefuroxime sodium	0	2.7	0	0
Cefuroxime axetil	3.2	5.3	4.5	2.2
Co-trimoxazole	16.1	16	9.1	11.1
Nalidixic acid	9.7	6.7	0*	8.9
Ciprofloxacin	6.5	2.7	0	4.4
Mecillinam	25.8	21.3	0	0
Nitrofurantoin	N/A ^c^	N/A ^c^	40.9	33.3
Fosfomycin	0	0	0	2.2
Cefotaxime	3.2	3.2	0	0
Ceftazidime	6.5	5.3	0	0
Gentamicin	0	2.7	0	
Netilimicin	0	0	0	2.2
Amikacin	0	0	4.5	4.4
Piperacillin-tazobactam	0	0	0	0
Ticarcillin-clavulanate	0	0	0	0
Imipenem	0	0	0	0
Cefditoren	3.2	2.7	0	2.2

^a^ CLSI 2011 breakpoints of susceptibility were applied; ^b^ Acute Uncomplicated Cystitis, ^c^ not applicable, , *P ≤ 0.05.

### Risk factors for cefditoren non-susceptibility

In the total isolates yield (563 strains), cefditoren non-susceptibility was positively associated with the co-existence of non-susceptibility to other antimicrobials, with the exceptions of mecillinam, imipenem, fosfomycin, nitrofurantoin, gentamicin and netilmicin. Cefditoren MIC was significantly higher in nalidixic/ciprofloxacin non-susceptible strains. The same applied for ciprofloxacin MIC in cefditoren non-susceptible isolates (Table 5). These results were also true for the subgroup of isolates with available clinical information (data not shown). Univariate analysis of clinical information has demonstrated the following risk factors associated with Cefditoren non-susceptibility: the presence of fever (P = 0.016), history of urinary tract infection in the previous two weeks from sampling (P = 0.026), history of urinary tract infection in the previous three months (P = 0.012), use of a fluoroquinolone during the previous three months (P = 0.018) and use of a cephalosporin or a clavulanate fixed combination in the previous three months (P = 0.042). In the multivariate analysis only history of urinary tract infection in the previous two weeks and in the previous three months retained statistical significance (Table 6). Clinical and microbiological charecteristics of isolates with resistance in cefditoren are listed in Table 7.

**Table 5:** MIC50/90 distribution according to different resistance phenotypes

	**Cefditoren susceptible strains**	**Cefditoren non-susceptible strains**	**P**
Ciprofloxacin MIC50/MIC90 (mg/L)	0.012/0.032	0.19/32	<0.001
	Nalidixic acid susceptible strains	Nalidixic acid non-susceptible strains	P
Cefditoren MIC50/MIC90 (mg/L)	0.25/0.5	0.25/48	<0.001
	Ciprofloxacin susceptible strains	Ciprofloxacin non-susceptible strains	P
Cefditoren MIC50/MIC90 (mg/L)	0.25/0.5	0.5/64	<0.001

**Table 6:** Multivariate analysis of risk factors for cefditoren resistance

**Parameter**	***p *value**	**Odds Ratio**	**95% Confidence Interval**
Presence of fever	0.14	5.69	0.58-55.54
History of UTI in the previous 2 weeks	0.006	39.65	2.88-546.30
History of UTI in the previous 3 months	0.04	22.67	1.12-457.48
Fluoroquinolone use in the last 3 months	0.32	0.16	0.04-5.89
Cephalosporin or clavulanate fixed combination in the last 3 months	0.86	0.78	0.05-11.48

*UTI* urinary tract infection.

**Table 7:** Microbiological and clinical information of Cefditoren-resistant isolates

**Isolate**	**MIC Cefdi**	**CIP**	**AN**	**AMC**	**AM**	**Cefot CTX**	**CAZ**	**Cefur CXM**	**Cefal CF**	**FOS**	**GM**	**IMP**	**MEC**	**NA**	**NET**	**FT**	**TZP**	**TCC**	**SXT**	**Resistance Phenotype**	**Clinical details available**
**BA 356*E. coli***	1	S	S	I	R	S	S	S	S	S	S	S	S	S	S	S	S	S	S	TEM-1 hyperproduction	NA
**BA 425*E. coli***	1	S	S	S	R	S	S	S	S	S	S	S	S	S	S	S	S	S	R	TEM-1 + SXT^R^	75ys female AUC, doxycycline in the last 3mos
**BC 165*E. coli***	1	S	S	R	R	S	S	S	R	S	S	S	S	S	S	S	S	S	S	TEM-1 hyperproduction	25ys female UTI, Hx of UTI in the last 2wks
**BC 211*E. coli***	1	R	S	I	R	S	S	S	R	S	S	S	S	R	S	S	S	S	R	TEM-1 hyperproduction + SXT^R^ + cross resistance to all fluoroquinolones	81ys female AB with diabetes mellitus and nephrolithiasis, Hx of UTI in the last 2ks and 3 mos, use of CIP in the last 3mos
**BTH 486*E. coli***	1	S	S	I	R	S	S	S	S	S	S	S	S	S	S	S	S	S	R	TEM-1 hyperproduction + SXT^R^	NA
**BM 538*E. coli***	1	S	S	I	R	S	S	S	R	S	S	S	S	S	S	S	S	S	S	TEM-1 hyperproduction	NA
**PER 41ii*E. coli***	1	S	S	S	R	S	S	S	S	S	S	S	S	S	S	S	S	S	S	TEM-1	45ys male AB, Hx of UTI in the last year
**BTH 229*Klebsiella*spp**	2	S	S	S	R	S	S	S	S	S	S	S	S	S	S	I	S	S	R	SHV-1 natural + FT^R^ + SXT^R^	NA
**BP 129*E. coli***	2	-	S	R	R	S	S	I	R	R	S	S	S	S	S	R	S	S	S	TEM-1 hyperproduction + FT^R^ + FOS^R^	NA
**P 72*Klebsiella*spp**	2	R	S	S	R	S	S	S	S	S	S	S	S	R	S	R	S	S	R	SHV-1 + FT^R^ + SXT^R^ + cross resistance to all fluoroquinolones	80ys female AB with folley catheter, Hx of UTI in the last 2wks and 3mos, use of cefaclor and TZP in the last 3 months
**BK 319*E. coli***	4	R	S	I	R	S	S	S	S	S	S	S	S	R	S	S	S	S	R	TEM-1 hyperproduction + SXT^R^ + cross resistance to all fluoroquinolones	18ys female AUC, Hx of UTI in the last 3mos
**BA 569*E. coli***	16	S	S	S	R	R	S	R	R	S	S	S	S	S	S	S	S	S	R	ESBL CTX-type + SXT^R^	NA
**BTH 470*E. coli***	32	S	S	S	R	R	S	R	R	S	S	S	S	R	S	S	S	S	S	ESBL CTX-type + Resistance quinolones Nal^R^	40ys female AUC, Hx of UTI in the last year
**BM 593*E. coli***	32	S	S	S	R	R	S	R	R	S	S	S	S	R	S	S	S	S	R	ESBL CTX-type + SXT^R^ + Resistance quinolones Nal^R^	NA
**BA 524*Klebsiella*spp**	64	R	S	S	R	R	R	R	R	S	S	S	R	R	S	S	S	S	S	ESBL + Mecilinam^R^ + Cross resistance to all fluoroquinolones	NA
**BM 463*P. mirabilis***	64	S	S	R	R	S	S	S	R	S	S	S	S	S	S	R	S	S	S	AmpC plasmidic + natural FT^R^	56ys female, fever-frequency-dysuria-pyuria, use of cefaclor in the last 3 mos
**BM 465*P. mirabilis***	64	S	S	R	R	S	S	S	R	S	S	S	S	S	S	R	S	S	S	AmpC plasmidic + natural FT^R^	36ys female AUC, history of UTI in the last 3mos and use of CIP
**BM 466*P. mirabilis***	64	S	S	R	R	S	S	S	R	S	S	S	S	S	S	R	S	S	S	AmpC plasmidic + natural FT^R^	NA
**BA 315*E. coli***	128	S	S	I	R	R	S	R	R	S	S	S	S	R	S	S	S	I	I	ESBL CTX-type + SXT^R^ + Resistance quinolones Nal^R^	80ys female AB with folley catheter, Hx of UTI in the last 3mos, use of SXT and TZP in the last 3mos, recent hospital admission
**GEM 167*E. coli***	128	S	S	I	R	R	S	R	R	S	S	S	S	S	S	S	S	S	R	ESBL CTX-type + SXT^R^	NA
**GEM 250*E. coli***	128	R	S	S	R	R	S	R	R	S	S	S	S	R	S	S	S	S	R	ESBL CTX-type + SXT^R^ + Cross resistance to all fluoroquinolones	68ys female, fever-pyuria, diabetes mellitus and nephrolithiasis, Hx of UTI in the last 2wks and use of CIP, Hx of recent hospital admission
**GEM 315*E. coli***	128	S	R	S	R	R	R	R	R	R	S	S	R	S	S	R	I	S	R	ESBL + SXT^R^ + FT^R^ + FOS^R^	NA
**TZ 250*E. coli***	128	R	R	R	R	R	R	R	R	S	R	R	R	R	R	R	R	R	R	MBL (XDR) + Cross resistance to all fluoroquinolones	NA

CIP: Ciprofloxacin, AN: Amikacin, AMC: Amoxicillin / clavulanic acid, AM: Ampicillin, CTX: Cefotaxime, CAZ: Ceftazidime, CXM: Cefuroxime, CF: Cephalothin, FOS: Fosfomycin, GM: Gentamicin, IMP: Imipenem, MEC: Mecillinam, NA: Nalidixic acid, NET: Netilmicin, FT: Nitrofurantoin, TZP: Piperacillin / tazobactam, TCC: Ticarcillin / clavulanate, SXT: Trimethoprim / sulfamethoxazole. S: Sensitive, I:Intermediate susceptibility, R: Resistant, NA: not available, AUC: acute uncomplicated cystitis, CUTI: complicated urinary tract infection, AB: asymptomatic bacteriuria, Hx of UTI: history of urinary tract infection.

## Discussion

Cefditoren is a rapidly bactericidal antibiotic as demonstrated by previous in vitro studies [7-9]. Clinical data on its use in urinary tract infections are currently lacking whereas microbiological data against uropathogens are scarce [13,14]. Currently there are not established susceptibility breakpoints for cefditoren against Gram-negative rods; for this reason we used as tentative breakpoint of susceptibility the threshold of ≤1 mg/L, according to recently published evidence as well as the most recently proposed clinical breakpoints by EUCAST, setting susceptibility of the 3^rd^ generation cephalosporins against Enterobacteriaceae at ≤1 mg/L [13-15].

This is among the first studies which report antimicrobial susceptibility data of cefditoren in comparison to other commonly used antimicrobials from a large population-based surveillance study of outpatients with UTIs. According to the data presented herein, cefditoren had the lowest rate of resistance among the tested orally-available antibiotics after fosfomycin- which is not marketed in our country in the last 10 years. In vitro activity of cefditoren in our population of uropathogens was in accordance with data from Spain [14], whereas a study from Korea reported higher MIC90 (16 mg/L) compared to our data (0.5 mg/L) [13]. As reported previously, cefditoren was not active against ESBL-producing strains [9,13], however its activity against TEM/hyperproducers was more variable compared to previous reports although the majority of the strains (63.6%) were inhibited in clinically achievable concentrations [9].

Resistance to cefditoren was associated in our study with resistance to nalidixic acid- and resistance to ciprofloxacin. These observations are in accordance with data from Korea, a country with high levels resistance to ciprofloxacin among uropathogens (30.3% in the study by Ko et al.) [13]. On the other hand, no association of resistance to cefditoren was found with mecillinam, fosfomycin, nitrofurantoin and aminoglycosides, indicating that their use as first line treatment options in community acquired UTIs as recommended by local guidelines would not affect the susceptibility of cefditoren. This observation was also confirmed in a recent large European multicenter study [17]. In both ECO⋅SENS studies, resistance to any antimicrobial studied was markedly higher in isolates resistant to any other antimicrobial, irrespective of whether or not the antibiotics belonged to the same class [17,18]. This has been partially attributed to a pool of resistance in aminopenicillins, folate inhibitors and fluoroquinolones among *E.coli* in the community and the long established plasmid- mediated resistance to ampicillin and to trimethoprim/ sulfamethoxazole [17].

The accompanying questionnaire enabled us to classify UTIs as “complicated” or “uncomplicated” and to elucidate risk factors for resistance to cefditoren beyond the well-recognized use of an antibiotic of the same class in the previous 15 days-3 months [6,19,20]. The presence of fever indicating complicated UTI, previous use of a cephalosporin or a fixed combination of clavulanate or a quinolone for any reason and recent history of UTI in the last two weeks and three months were elucidated in the univariate analysis as risk factors for cefditoren resistance; however only the history of UTI retained statistical significance in the multivariate analysis, with an Odds Ratio of 39.65 for the preceding two weeks and 22.67 for the preceding two months. Cephalosporins are not currently indicated as first line antibiotics in acute uncomplicated cystitis, not only because other classes of antibiotics provide more convenient schemes of treatment, but also for epidemiological reasons [3,21]. However, an emerging trend of resistance in cotrimoxazole is documented in several regions [2,6,13,14]. IDSA and several other national guidelines advise against the empirical use of cotrimoxazole in settings with known resistance above the threshold of 20% [3,20,21]. In our country, data acquired through a big population-based surveillance revealed a resistance rate of 19.2% for AUC *E. coli* strains [10]. According to these recently published data from our group, the most potent in vitro antibiotics available for oral use in AUC in our community were fosfomycin, mecillinam, cefuroxime axetil, ciprofloxacin and amoxicillin/clavulanic acid with resistance rates of 1.6%, 3.4%,1.7%, 2.2% and 5.2% respectively. Interestingly, in this study cefditoren and oral cefuroxime displayed co-resistance only in isolates harboring ESBL or MBL mechanism of resistance (Table 7), although their overall susceptibility rates in the studied population were comparable (97.1% versus 94.1% respectively). Currently available data do not permit to adopt for cefuroxime the same risk factors elucidated for cefditoren but it is important to note that compared to other countries, cephalosporins of 2nd and 3rd generation and amoxicillin/clavulanic acid retain susceptibility among common uropathogens in UTIs in our community [2,10,17,18]. As mentioned above, fosfomycin trometamol is no longer available in the Greek market. On the other side, collateral damage with the use of quinolones as well an alarming percentage of first step mutants among uropathogens in both uncomplicated (6%) and complicated infections (12.7%) [10] has prompted severe restriction policies against their indiscriminate use in the Greek community; this is also true in many countries [22,23]. Furthermore the between the ECO⋅SENS I (1999–2000) and ECO⋅SENS II (2007–2008) studies ciprofloxacin resistance almost doubled in Greece [17,18], mandating quinolone sparing policies in our community.

A certain weakness of this study is the lack of clinical data for almost one third of the studied isolates. However the lack of significant differences in resistance rates against all antibiotics tested between the populations with and without clinical information, as well as the large and representative sample of our study argues in favor of an extended applicability of the conclusions in our community. Furthermore the addition of a pool of isolates without clinical data allowed for a simulation of real life treatment scenario of an outpatient with UTI. Another possible weakness is the lack of clinical therapeutic data, which would enable us to detect therapeutic failure in the community with currently used agents, but this was beyond the design and the scope of the current study.

Certainly no strong argument exists for the use of cefditoren as first line option in acute uncomplicated UTIs since other treatment options mentioned above retain good activity. However many UTIs treated in the community setting are not episodes of acute uncomplicated cystitis mandating concentrations in the upper urinary tract; patients are often pretreated with other first-line classes of antibiotics; apart from recent antibiotic use patients with complicated urinary tract infections treated as outpatients frequently bear risk factors for resistance to multiple classes of antibiotics [3,20]. Cefditoren could represent a treatment option in several cases among the above-mentioned patient/risk groups. According to our results, cefditoren could also be used in our community as a switch to oral treatment of patients with UTIs and risk factors for resistance to 1^st^ line options, initially treated with a parenteral antibiotic. A strong argument for the selection of cefditoren could be the lack of cross-resistance with aminoglycosides, mecillinam, nitrofurantoin and fosfomycin.

## Conclusions

In summary, cefditoren was the most potent in vitro antibiotic available for oral use in our country, against all three major pathogens causing UTIs in the outpatient setting. A potential was demonstrated for its use as empirical treatment in outpatients with no recent history of UTI, as an alternative to first line antimicrobials or in patients with risk factors for resistance to currently indicated first treatment options. Clinical studies are warranted to clearly define its possible role in the treatment of UTIs in outpatients.

## Competing interests

All authors declared no competing interests related to this manuscript.

## Authors’ contributions

DH carried out the in vitro procedures and input of data, and helped to draft the manuscript. GP: Participated in the design of the study, drafting and refinement of the manuscript, interpretation of data and also carried out in vitro procedures. IK: Management of the database, input and evaluation of data, statistical analysis, drafting of the manuscript. NL: Carried out laboratory procedures with determination of in vitro susceptibilities and input of data. MS: Carried out laboratory procedures and evaluation of data. ID: Carried out laboratory procedures and evaluation of data. GKN: Performed the statistical analysis. EL: Carried out laboratory work and evaluation of in vitro data. HG: Conceived the study, participated in the study design, drafting and refinement of the manuscript and interpretation of data. All authors have read and approved the final manuscript.

## Pre-publication history

The pre-publication history for this paper can be accessed here:

http://www.biomedcentral.com/1471-2334/12/228/prepub
